# Genome-wide identification of *PTI1* family in *Setaria italica* and salinity-responsive functional analysis of *SiPTI1–5*

**DOI:** 10.1186/s12870-021-03077-4

**Published:** 2021-07-03

**Authors:** Yongguan Huangfu, Jiaowen Pan, Zhen Li, Qingguo Wang, Fatemeh Mastouri, Ying Li, Stephen Yang, Min Liu, Shaojun Dai, Wei Liu

**Affiliations:** 1 Key Laboratory of Saline-alkali Vegetation Ecology Restoration (Northeast Forestry University), Ministry of Education, College of Life Sciences, Northeast Forestry University, Harbin, 150040 Heilongjiang China; 2 Shandong Academy of Agricultural Sciences, Jinan, 250100 Shandong China; 3 Bota Bioscience, 325 Vassar st. Suite 2a, Cambridge, MA 02139 USA; 4 Institute for Bioscience and Biotechnology Research, 9600 Gudelsky Dr, Rockville, MD 20850 USA; 5 Shandong Agriculture and Engineering University, Jinan, 250100 Shandong China; 6 Development Center of Plant Germplasm Resources, College of Life Sciences, Shanghai Normal University, Shanghai, 200234 China; 7 College of Life Sciences, Shandong Normal University, Jinan, 250014 Shandong China

**Keywords:** Foxtail millet (*Setaria italica*), Pto-interacting 1 genes (*PTI1s*), Expression pattern, Functional identification, Salt stress

## Abstract

**Background:**

PTI1 (Pto-interacting 1) protein kinase belongs to the receptor-like cytoplasmic kinase (RLCK) group of receptor-like protein kinases (RLK), but lack extracellular and transmembrane domains. PTI1 was first identified in tomato (*Solanum lycopersicum*) and named *SlPTI1*, which has been reported to interact with bacterial effector Pto, a serine/threonine protein kinase involved in plant resistance to bacterial disease. Briefly, the host PTI1 specifically recognizes and interacts with the bacterial effector AvrPto, which triggers hypersensitive cell death to inhibit the pathogen growth in the local infection site. Previous studies have demonstrated that *PTI1* is associated with oxidative stress and hypersensitivity.

**Results:**

We identified 12 putative *PTI1* genes from the genome of foxtail millet (*Setaria italica*) in this study. Gene replication analysis indicated that both segmental replication events played an important role in the expansion of *PTI1* gene family in foxtail millet. The *PTI1* family members of model plants, i.e. *S. italica*, Arabidopsis (*Arabidopsis thaliana*), rice (*Oryza sativa*), maize (*Zea mays*), *S. lycopersicum*, and soybean (*Glycine max*), were classified into six major categories according to the phylogenetic analysis, among which the *PTI1* family members in foxtail millet showed higher degree of homology with those of rice and maize. The analysis of a complete set of *SiPTI1* genes/proteins including classification, chromosomal location, orthologous relationships and duplication. The tissue expression characteristics revealed that *SiPTI1* genes are mainly expressed in stems and leaves. Experimental qRT-PCR results demonstrated that 12 *SiPTI1* genes were induced by multiple stresses. Subcellular localization visualized that all of foxtail millet *SiPTI1s* were localized to the plasma membrane. Additionally, heterologous expression of *SiPTI1–5* in yeast and *E. coli* enhanced their tolerance to salt stress.

**Conclusions:**

Our results contribute to a more comprehensive understanding of the roles of PTI1 protein kinases and will be useful in prioritizing particular PTI1 for future functional validation studies in foxtail millet.

**Supplementary Information:**

The online version contains supplementary material available at 10.1186/s12870-021-03077-4.

## Background

PTI1 (Pto-interacting 1) protein kinase belongs to the receptor-like cytoplasmic kinase (RLCK) group of receptor-like protein kinases (RLK), but lack extracellular and transmembrane domains [1, 2]. In plants, PTI1 play an important role in plant defense against bacterial pathogens. It was first identified in tomato and was demonstrated to specifically recognize and interact with the AvrPto effector protein injected into the plant cells by the pathogenic bacteria, thereby triggering the downstream defense response [3].

PTI1 generally contains a kinase domain consisting of 250 to 300 amino acid residues [4], and possess characteristic domains of STKc_IRAK, Pkinase_Tyr, STYKc, and SPS1 [5, 6]. In recent years, *PTI1* genes had been widely identified in many species such as tomato (*Solanum lycopersicum*) [3, 7], Arabidopsis (*Arabidopsis thaliana*) [1, 8], maize (*Zea mays*) [9, 10], soybean (*Glycine max*) [11, 12], cucumber (*Cucumis sativus*) [13] and rice (*Oryza sativa*) [14].

*PTI1* genes in different species and subtypes are involved in different processes. In Arabidopsis, PTI1–1, PTI1–2, PTI1–3, PTI1–4 and PTI1–5 were reported to interact with protein kinase OXIDATIVE SIGNAL INDUCIBLE1 (OXI1) and are phosphorylated by OXI1 in response to phosphatidic acid (PAs), H_2_O_2_, flg22, and xylanase [8, 15]. Moreover, *PTI1–2*/*PTI1–4* responds to oxidative stress via *OXI1-PTI1–2*/*PTI1–4* pathway [1, 8]. Abiotic stress activated *PTI1–2* also enhances the expression of reactive oxygen species (ROS) stress-responsive genes [1]. OXI1-PTI1 is also involved in the activation of the MAPK signaling pathway, which in turn responds to oxidative and biotic stresses [8, 16]. *AtPTI1–5* knockout greatly affects the growth of pollen tubes resulting in male gametophyte sterility [15]. Tomato SlPTI1 interacts with and is activated by Pto, which regulates downstream signal transduction upon pathogen invasion [3, 17]. There are four members of the *PTI1s* in maize, which *ZmPTI1a* is involved in pollen propagation [9]. The *ZmPTI1a* hetero-overexpressed Arabidopsis lines showed enhanced salt stress tolerance, with higher fresh and dry weight compared to wild type plants [10]. Overexpressing cucumber *CsPTI1-L* in tobacco could enhance salt tolerance via up-regulation of multiple resistance-related genes [13]. Overexpression of *OsPTI1* increases rice resistance to fungal invasion [14].

Foxtail millet (*Setaria italica*) was domesticated in neolithic China approximately 8700 years ago and has been regarded as an important dietary staple food in China for millennia [18, 19]. It possesses attractive qualities, such as small diploid genome (~ 510 Mb) [20], lower repetitive DNA, short life cycle, and C4 photosynthesis [21, 22]. These characteristics promote it as a model crop for exploring basic biology processes, such as plant architecture, physiology and genome evolution [23, 24]. At the same time, the stresses and barren tolerance characteristics of foxtail millet make them reduced the dependence on synthetic fertilizers, pesticides, herbicides, and insecticides [25]. And millet cultivation could decrease the over-reliance on the major cereals that are limited in number worldwide [23]. Especially during the hard time of COVID-19 pandemic around the world, the strategic roles of foxtail millet in stabilizing grain production, ensuring the global economy and people’s livelihood are attracted more and more attentions worldwide [26, 27]. Analysis of stress resistance mechanisms and quality traits of foxtail millet are important for the development of modern foxtail millet germplasms or cultivars. With the rapid development of molecular biology, the whole genome of foxtail millet has been sequenced and published, which enables better understanding of the stress response and molecular regulatory mechanisms of this crop plant [28, 29].

*PTI1* gene family of foxtail millet is not yet been identified. In our previous transcriptome analysis of salt stress in foxtail millet, a stress induced gene of Seita.5G023100.1 with unknown function were identified [30]. JGI/NCBI BLAST sequence analysis showed that it was a putative PTI1 protein kinase. Considering that PTI1 proteins participate in a variety of stress defense responses in several plant species such as tomato [3, 7], Arabidopsis [1, 8], maize [9, 10], and no PTI1 was identified in foxtail millet up to now. The systematic analysis of *PTI1* gene family was carried out in this study, and 12 *PTI1* genes were identified. Their chromosomal locations and protein structures were predicted and analyzed. The expression patterns of 12 *SiPTI1* genes were analyzed by quantitative real-time PCR (qRT-PCR). Our results showed that most *SiPTI1* genes were differentially expressed in response to salt stress and oxidative stress. A key gene *SiPTI1–5* that may be associated with salt stress was selected for further studies. Overexpression of *SiPTI1–5* in yeast and *Escherichia coli* (*E. coli*) enhance their tolerance to salt stress.

These results could deepen our understanding of the characteristics and functions of *PTI1* genes in foxtail millet, and also assist to identifies potential abiotic stress-responsive genes for improving foxtail millet and other crop species. In addition, this study is the first systematic report on the *PTI1* gene family in plants, which will also provide reference for the subsequent systematic study on the function of *PTI1* genes in foxtail millet. At the same time, it also provides reference for the study of *PTI1* genes family in other species.

## Results

### *SiPTI1s* identification and annotation in foxtail millet

Our transcriptome analysis of salt stress in foxtail millet revealed an over-expressed gene (Seita.5G023100.1) with unknown function [30]. JGI/NCBI BLAST sequence analysis showed that it was a putative PTI1 protein kinase. In view of the fact that PTI1 proteins participate in a variety of stress defense responses and no *PTI1* was previously identified in foxtail millet, we decided to further analyze the *PTI1* gene family in foxtail millet to identify those responsive to salt stress and explore their application in crop improvement.

**Fig. 1:**
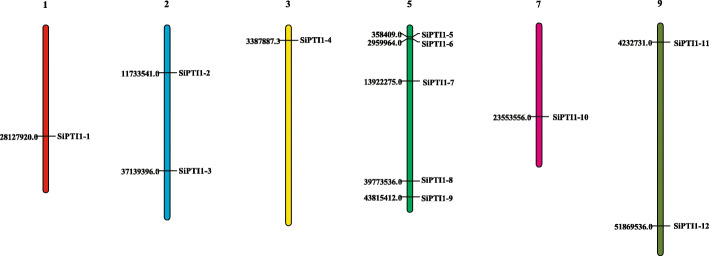
Distribution of 12 *SiPTI1* genes onto the nine foxtail millet chromosomes. Localization of the foxtail millet *PTI1* genes on the foxtail millet chromosomes. Chromosomal distances are given in bp

In this study, a total of 12 putative *PTI1* genes were identified in foxtail millet via genome-wide analysis (Table 1, Additional file 1). The genes were named *SiPTI1–1* to *SiPTI1–12* according to their location on the chromosome. Foxtail millet has 9 chromosomes, ranging from 35.9 Mb (chromosome 6) to 58.9 Mb (chromosome 9). The physical map positions of the 12 *SiPTI1* genes in the 9 chromosomes of foxtail millet are presented in Fig. 1. The specific location of each *SiPTI1* gene on the chromosome was provided in the Additional file 4. However, the distribution of *SiPTI1s* on chromosomes was uneven, with five genes located on chromosome 5 (*SiPTI1–5*, *SiPTI1–6*, *SiPTI1–7*, *SiPTI1–8*, and *SiPTI1–9*) and only one gene located on chromosome 1, chromosome 3 and chromosome 7, respectively. Interestingly, chromosome 9 is the longest, but only two *SiPTI1s* are located on it (*SiPTI1–11* and *SiPTI1–12*). Therefore, there was no positive correlation between the chromosome length and the number of *PTI1* genes.

**Table 1:** The identification of PTI1 members in *Setaria italica*

Gene name	Gene ID	Protein length (aa)	MW (Da)	pI	Prediction of protein Subcellular location
*SiPTI1–1*	Seita.1G201600.1	364	39,048	8.43	plasmamembrane
*SiPTI1–2*	Seita.2G116400.1	369	40,816.1	6.44	plasmamembrane
*SiPTI1–3*	Seita.2G271300.1	366	40,364.7	6.84	plasmamembrane
*SiPTI1–4*	Seita.3G053500.1	362	39,229.4	7.89	plasmamembrane
*SiPTI1–5*	Seita.5G023100.1	727	80,955.8	8.38	plasmamembrane
*SiPTI1–6*	Seita.5G030900.1	428	46,446.6	9.15	plasmamembrane
*SiPTI1–7*	Seita.5G154400.1	426	46,095.4	9.26	plasmamembrane
*SiPTI1–8*	Seita.5G358000.1	395	43,452	6.01	plasmamembrane
*SiPTI1–9*	Seita.5G415300.1	387	42,967.9	7.8	plasmamembrane
*SiPTI1–10*	Seita.7G147800.1	388	42,733.1	7.38	plasmamembrane
*SiPTI1–11*	Seita.9G072200.1	364	39,016	8.43	plasmamembrane
*SiPTI1–12*	Seita.9G478500.1	366	38,980.2	8.84	plasmamembrane

Their domains were further confirmed by the three databases of SMART, NCBI CDD and Pfam. Gene classification and detailed annotation are listed in Table 1. The predicted SiPTI1s protein sequences ranged from 362 amino acids (SiPTI1–4) to 727 amino acids (SiPTI1–5), and the corresponding molecular weights varied from 38.9802 to 80.9558 kDa. The predict pI varied from 6.01 to 9.26. Little differed among the 12 SiPTI1 proteins except SiPTI1–5, generally, the length of PTI1 protein kinase was about 300–400 amino acids, while SiPTI1–5 encodes 727 amino acids, and the highest molecular weight was about 81 kDa.

### Phylogenetic analysis of *SiPTI1s* with *PTI1s* of other plant species

**Fig. 2:**
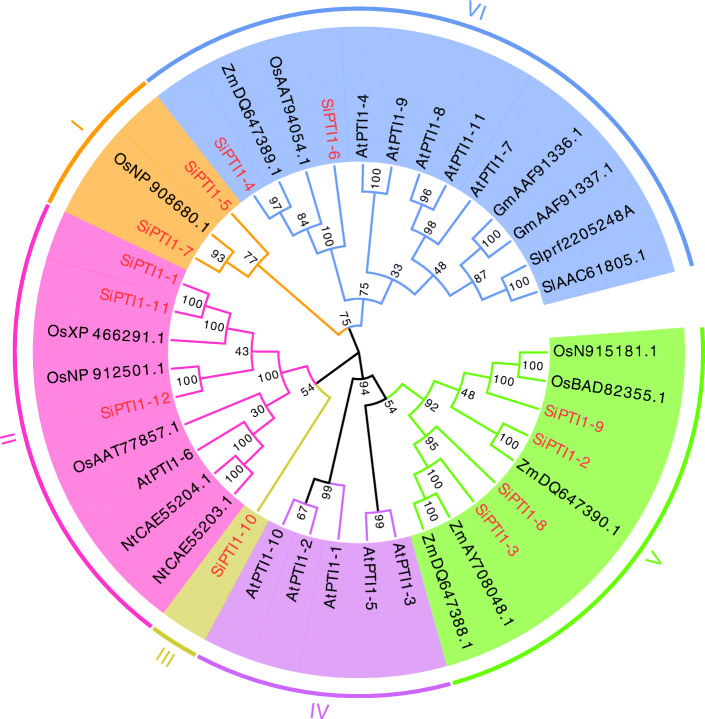
Phylogenetic of the PTI1 proteins in different species. Phylogenetic analysis was based on 45 PTI1 protein sequences. Species abbreviations are as follows. At: *A. thaliana*; Os: *O. sativa*; Zm: *Z. mays*; Sl: *S. lycopersicum*; Gm: *G. max*; Nt: *N. tabacum*. Multiple sequence alignments of PTI1 amino-acid sequences were performed using ClustalX, and the phylogenetic was constructed using MEGA7 by the maximum likelihood method and 1000 bootstrap replicates. The tree was divided into six phylogenetic subgroups, designated I-VI. Letters outside of the tree indicate the defined groups

The *PTI1* genes have been identified in many plant species in the recent years. Based on the publicly available information and the degree of relatedness, we chose *PTI1* genes from *A. thaliana* (At), *O. sativa* (Os), tobacco (*Nicotiana tabacum*) (Nt), *Z. mays* (Zm), *S. lycopersicum* (Sl), and *G. max* (Gm) to construct the phylogenetic trees with the *PTI1* genes of *S. italica* (Si). As shown in (Fig. 2, Additional file 2), the phylogenetic analysis suggested that all *PTI1* genes could be grouped into six classes and each SiPTI1 protein sequence was highly similar to their homologues in other plant species. Since a good number of the internal branches were observed to have high bootstrap values. The phylogenetic tree also revealed that the majority of foxtail millet *SiPTI1* families distribution predominates with species bias, they are more closely related to those in grass species (rice and maize), in contrast, they are relatively distant relatives of the dicotyledonous Arabidopsis.

### Gene structure, motif patterns analysis of *PTI1* genes in foxtail millet

**Fig. 3:**
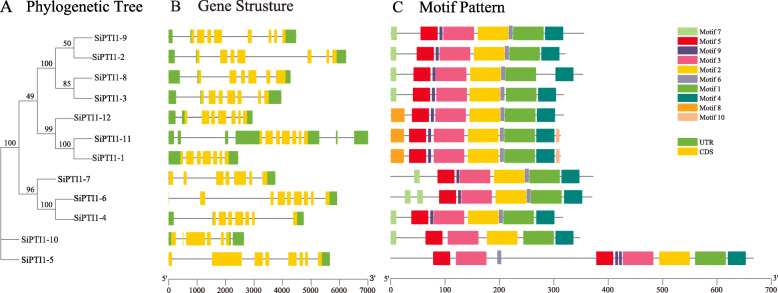
Phylogenetic relationships, gene structure and architecture of conserved protein motifs in *PTI1* genes from foxtail millet. The phylogenetic tree was constructed based on the full-length sequences of foxtail millet PTI1 proteins using MEGA7 software (**A**). Exon-intron structure of foxtail millet *PTI1* genes. Green boxes indicate untranslated 5′- and 3′-regions; yellow boxes indicate exons; black lines indicate introns (**B**). The motif composition of foxtail millet PTI1 proteins. The motifs, numbers 1–10, are displayed in different colored boxes (**C**). The sequence information for each motif is provided in Additional file 2. The length of protein can be estimated using the scale at the bottom

To explore the structural diversity of *SiPTI1s*, the distribution of exon-intron structure was analyzed and mapped in the phylogenetic tree. As shown in Fig. 3B, two *PTI1* genes (*SiPTI1–9* and *SiPTI1–5*) contained seven introns, *SiPTI1–8* and *SiPTI1–10* had five introns and *SiPTI1–6* had eight introns. The rest of *SiPTI1* genes had six introns. Exon-intron structural analysis indicated that members of some *PTI1* subfamilies have similar exon-intron structures. Similar results were also found in maize [31] and other studies.

The motif patterns among *SiPTI1*s were investigated (Fig. 3 C and Additional file 3). A total of 10 motifs were discovered and 5 of them were found to be highly conserved. In addition, all of *SiPTI1*s contained motifs 1, 2, 3, 4 and 5. Except for *SiPTI1–10*, all of other *SiPTI1*s contain motifs 6 and 9. Furthermore, motif 8 was found in three of the *SiPTI1s* members (*SiPTI1–1*, *SiPTI1–11* and *SiPTI1–12*), while motif 10 was only presented in two members (*SiPTI1–1*, *SiPTI1–11*). Interestingly, the motif distribution of *SiPTI1–5* was different from other members of the family, in that motifs 3, 5, 9 appear twice each. Despite the difference of motif types between groups, members within the same group such as *SiPTI1–9* and *SiPTI1–2*, *SiPTI1–8* and *SiPTI1–3*, *SiPTI1–11* and *SiPTI1–1* tend to exhibit similar motif patterns (Fig. 3 A and C), which indicate functional similarity between them. Amino acid sequence analyses showed that the SiPTI1s contain the representative kinase domains, such as STKC_IRAK, Pkinase_Tyr, STYKc, and SPS1 (data not shown). As known that the catalytic domain of serine/threonine kinases contains 11 subdomains [31, 32], the pileup analysis also showed that the 12 SiPTI1 kinases also contained the conserved 11 subdomains like known *PTI1* gene of *SlPTI1* in tomato (Supplementary Fig. 2). In addition, when compared the SiPTI1s sequence of foxtail millet with the PTI1 sequences of maize and rice, we found that the catalytic domain of serine/threonine kinases also contains 11 subdomains, which were consistent with the results of SiPTI1s and SlPTI1 sequence analysis (Supplementary Fig. 3).

### *Cis*-acting elements and subcellular localization of *PTI1* genes in foxtail millet

**Fig. 4:**
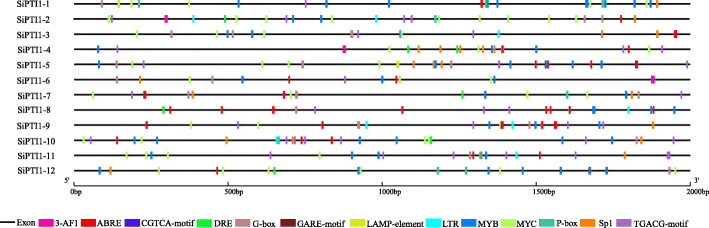
*Cis*-elements prediction in the 2.0 kb promoter region upstream from the start codon of *SiPTI1s*. The relative positions of *cis*-elements in each *SiPTI1* gene are marked by different-colored boxes

*Cis*-elements analysis showed that all *SiPTI1* genes promoter contained MYB, MYC and ABA-responsive (ABRE) elements. In addition, excepted for *SiPTI1–12*, both CGTCA-motif and TGACG-motif *cis*-elements were present in foxtail millet *PTI1* genes family (Fig. 4 and Additional file 5). In addition, 50% of the members had a low-temperature responsive element (LTR), and 75% contained a dehydration responsive element (DRE) (Fig. 4 and Additional file 5). Furthermore, *SiPTI1s* contained a large number of *cis*-elements regulatory elements involved in light response, such as Sp1, G-box, and AF-box. Gibberellin-responsive elements such as P-box and GARE-motif were also presented (Fig. 4 and Additional file 5).

**Fig. 5:**
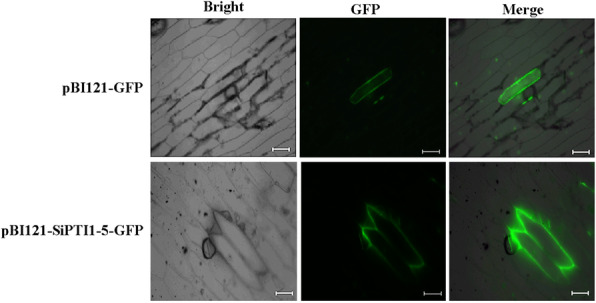
Subcellular localization of SiPTI1–5 protein. Fluorescent microscopic images of GFP and SiPTI1–5-GFP fusion protein in the onion epidermal cells (Bar = 100 μm)

Using five publicly available subcellular localization prediction tools we found that all of the SiPTI1s were predicted to localize in the plasma membrane (Table 1). To investigate the potential role of SiPTI1–5 a vital salt tolerance-related gene of PTI1 family in the foxtail millet, we examined the subcellular localization of SiPTI1–5 fused to GFP and GFP alone (as a control) in onion epidermal cells. When observed by confocal microscopy, the green fluorescent protein (GFP) fluorescence of SiPTI1–5-GFP was distributed on the plasma membrane in the onion cells (Fig. 5), which indicated that SiPTI1–5 was localized in the plasma membrane.

### Duplication and divergence rates of the *SiPTI1* genes

**Fig. 6:**
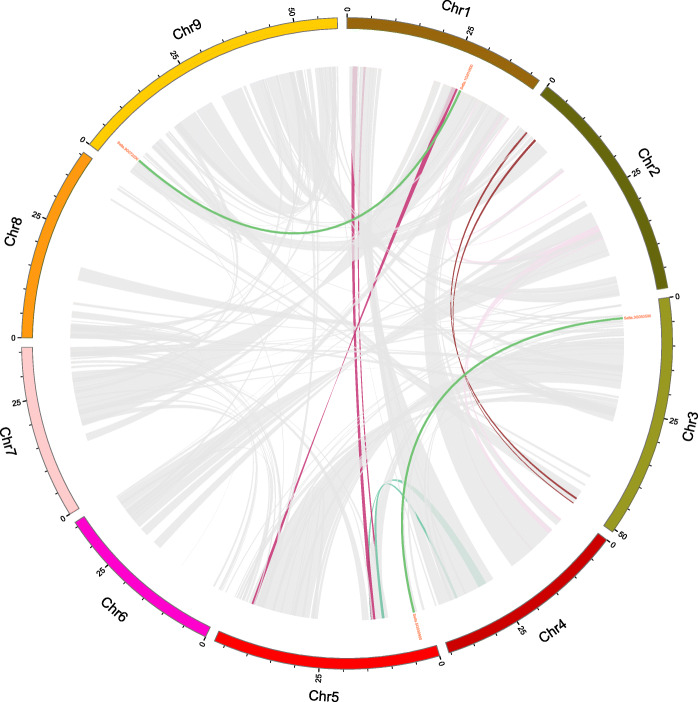
Schematic representations for the chromosomal distribution and interchromosomal relationships of foxtail millet *PTI1* genes. Gray or other color lines indicate all synteny blocks in the foxtail millet genome, and the dark green lines indicate duplicated *PTI1* gene pairs and the end of the line shows the ID number of the corresponding gene. The chromosome number is indicated at the bottom of each chromosome

Gene duplications, including segmental and tandem duplication, have long been considered as one of the main forces in the evolution and expansion of a gene family [33]. In addition, two pairs of segmentally duplicated genes were found within the *SiPTI1s* family (*SiPTI1–4*/*SiPTI1–6* and *SiPTI1–1*/*SiPTI1–11*) (Fig. 6 and Additional file 6). To further unveil the relationship between duplication events and natural selection, the Ka and Ks values of *SiPTI1s* in duplicated gene pairs were calculated, and the results of Ka/Ks values were found to be less than 1, suggesting that *SiPTI1* family has gone through purifying selection after gene duplications.

**Fig. 7:**
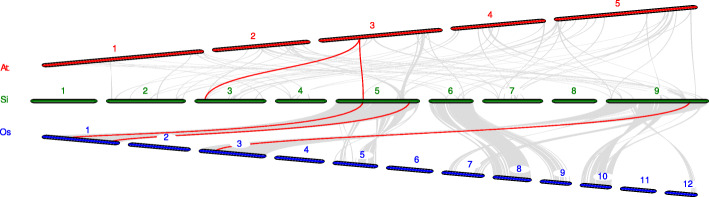
Synteny analysis of *PTI1* genes between foxtail millet and two representative plant species. Gray lines in the background indicate the collinear blocks within foxtail millet and other plant genomes, while the red lines highlight the syntenic *PTI1* gene pairs. The species names with ‘At’, ‘Os’, ‘Si’ indicate *Arabidopsis thaliana*, *Oryza sativa* and *Setaria italica*, respectively

To further infer the phylogenetic mechanisms of foxtail millet *PTI1* family, we constructed two comparative syntenic maps of foxtail millet associated with two representative species, including dicots (Arabidopsis) and monocots (rice) (Fig. 7 and Additional file 7). A total of 2 *SiPTI1* genes showed syntenic relationship with those in Arabidopsis. Moreover, we found that *SiPTI1–4* and *SiPTI1–7* present the same collinear gene (*AT3G17410*), three genes in rice had a colinear relationship with foxtail millet *SiPTI1s* (*SiPTI1–9*/*Os01t0899000*, *SiPTI1–7*/*Os01t0323100*, *SiPTI1–12*/*Os03t0226300*). In addition, compared to the Arabidopsis*,* the *PTI1s* gene of foxtail millet had more colinearity with the rice *PTI1* genes, and the colinearity between genomes was more abundant.

### Expression patterns of *SiPTI1*

**Fig. 8:**
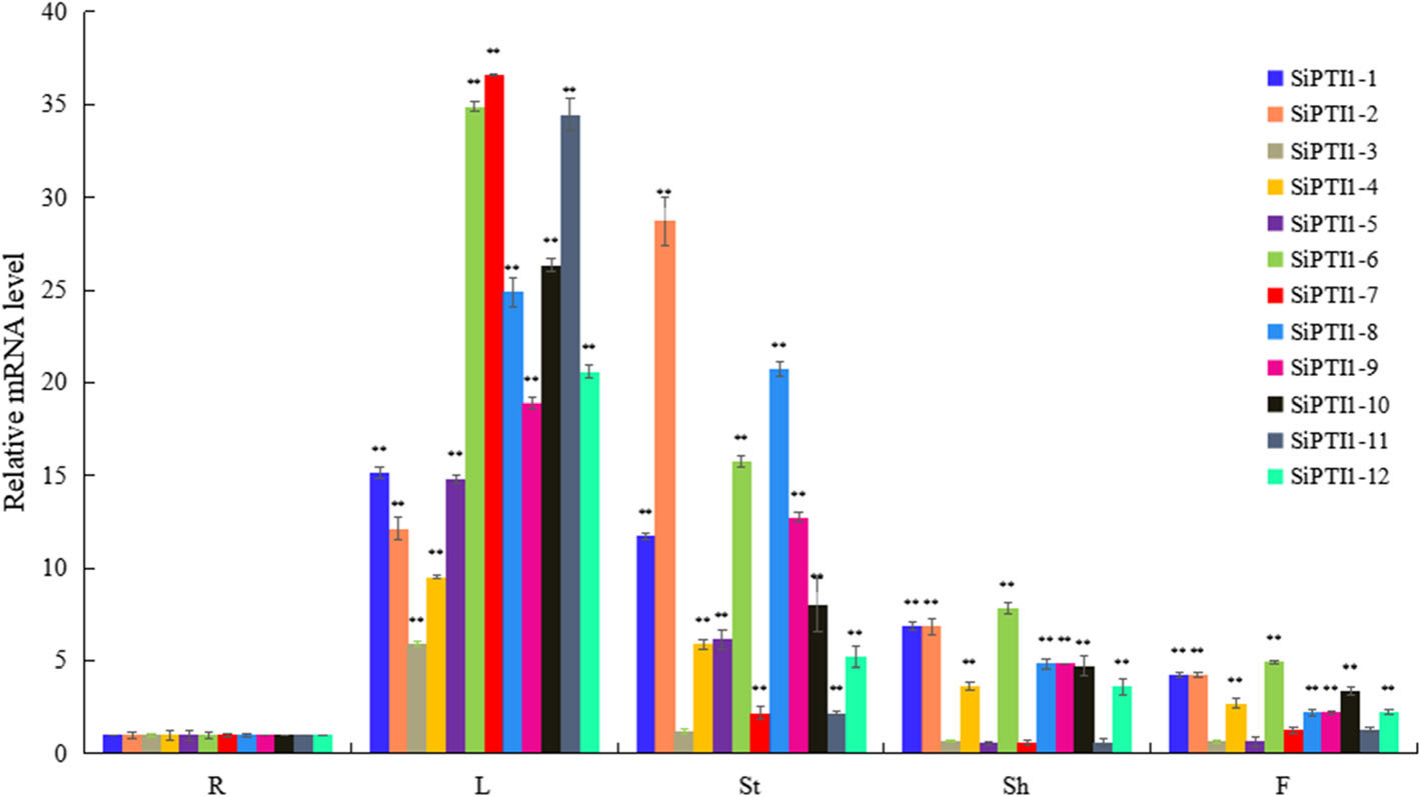
Expression profile analysis of *SiPTI1* genes in different foxtail millet tissues. Expression analysis of *SiPTI1s* by qRT-PCR. R, Roots; St, Stems; L, Leaves; Sh, Sheathes and F, Flowers. The values are the average of three biological repeats ± SD (standard deviation). Asterisks above bars denote a statistically significant difference by Duncan’s multi-range tests (*0.01 < *P* < 0.05, **P < 0.01)

To investigate the tissue-specific expressions of the 12 *SiPTI1* genes in foxtail millet, total RNA from roots, stems, leaves, sheaths and flowers were prepared and analyzed by qRT-PCR. As shown in Fig. 8 and additional file 9, the expressions of these *SiPTI1* genes were highest in the stems and leaves, followed by the expressions in the sheaths and flowers, and lowest in the roots. Except of *SiPTI1–3*, *SiPTI1–7*, and *SiPTI1–11*, the expression levels of *SiPTI1s* family members in stems were more than five-fold higher than those in roots, and the expression levels of all *SiPTI1* family members in leaves were also more than five-fold higher than that in roots. These results suggested that *SiPTI1* genes may perform an important function in the stems and leaves.

**Fig. 9:**
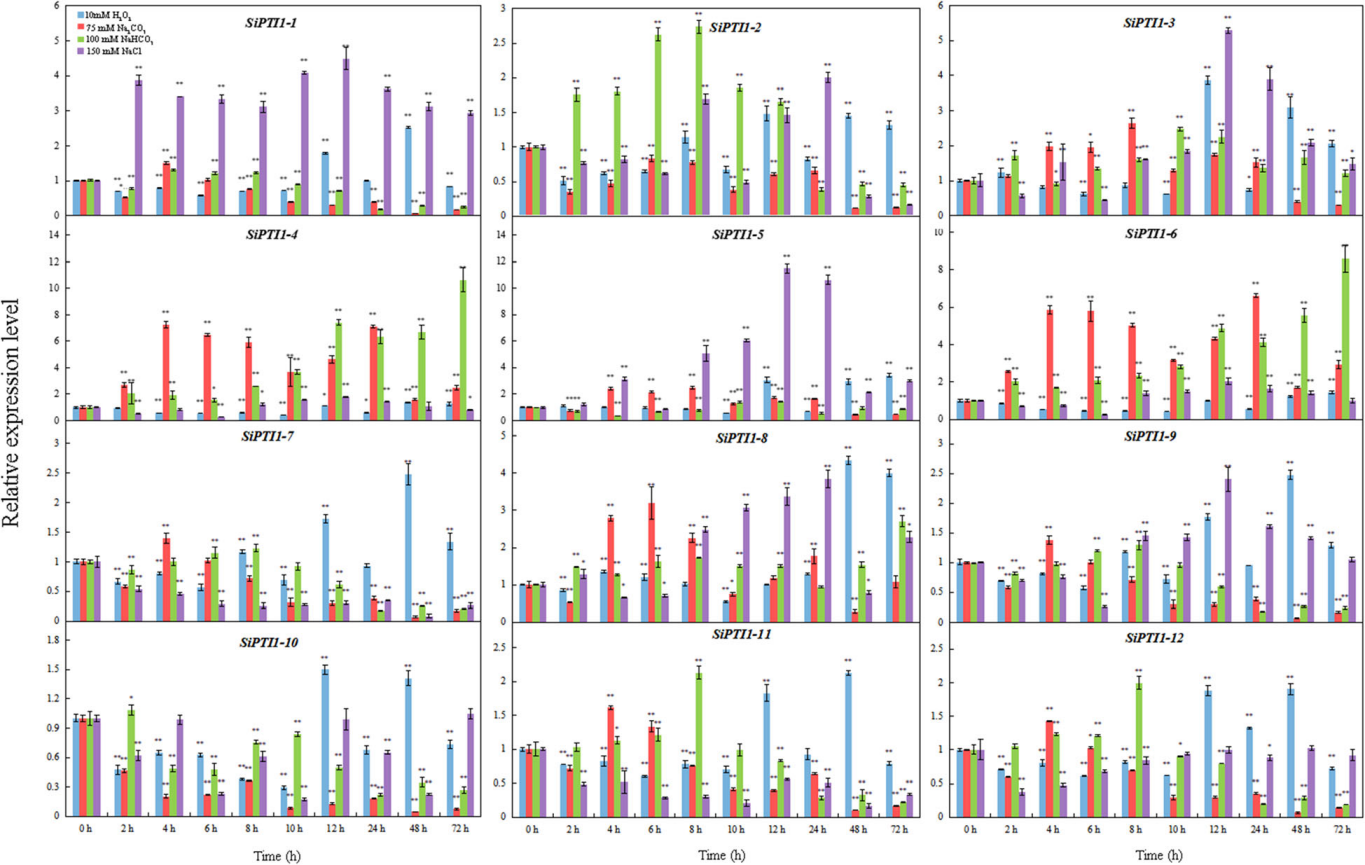
Expression profiles of *SiPTI1* genes in response to various abiotic stress treatments. The values are the average of three biological repeats ± SD (standard deviation). Asterisks above bars denote a statistically significant difference by Duncan’s multi-range tests (*0.01 < P < 0.05, **P < 0.01)

To further confirm whether the expression of *SiPTI1* genes were influenced by different abiotic stresses, we used qRT-PCR to monitor the expression patterns of the 12 *SiPTI1* genes in plants grown under different treatments namely salinity stress induced by treatment with NaCl, NaHCO_3_, Na_2_CO_3_, and oxidative stress induced by H_2_O_2_. As shown in Fig. 9 and Additional file 9, the expressions of most of the *SiPTI1* genes were responsive to abiotic stress treatment. The expression patterns of *SiPTI1* genes under NaCl-stress could classified into three categories. Firstly, fluctuation change, including *SiPTI1–2*, *SiPTI1–4*, *SiPTI1–6* and *SiPTI1–10*. The second, up-regulation expression trend, such as *SiPTI1–1*, *SiPTI1–3*, *SiPTI1–5*, *SiPTI1–8* and *SiPTI1–9*. Among them, the highest expression induced by NaCl was *SIPTI1–5.* In addition, the expression of *SiPTI1–5* reached peak when salt-stress treatment arrived at 12 h, which was about eleven-fold compare with control. The last one, down-regulation expression, including *SiPTI1–7*, *SiPTI1–11* and *SiPTI1–12*. Besides, under H_2_O_2_ treatment, most of *SiPTI1s* were induced at 12 h (Fig. 9). Under Na_2_CO_3_ treatment, most of *SiPTI1s* were induced at 4 h and 6 h, and then down-regulated after 8 h. Moreover, except of the up-regulated *SiPTI1*–*4*, *SiPTI1*–*6* and *SiPTI1*–*8*, other *SiPTI1s* were not significantly induced and/or down-regulated under Na_2_CO_3_ treatment, such as *SiPTI1*–*1* and *SiPTI1*–*10* (Fig. 9). In addition, under NaHCO_3_ stress, *SiPTI1*–*4* and *SiPTI1*–*6* were significantly induced (Fig. 9). Importantly, *SiPTI1–3* and *SiPTI1–5* were all up-regulated under the various stress conditions. Among them, *SiPTI1–5* was significantly induced up to 11.5-fold change under NaCl stress (Fig. 9).

**Fig. 10:**
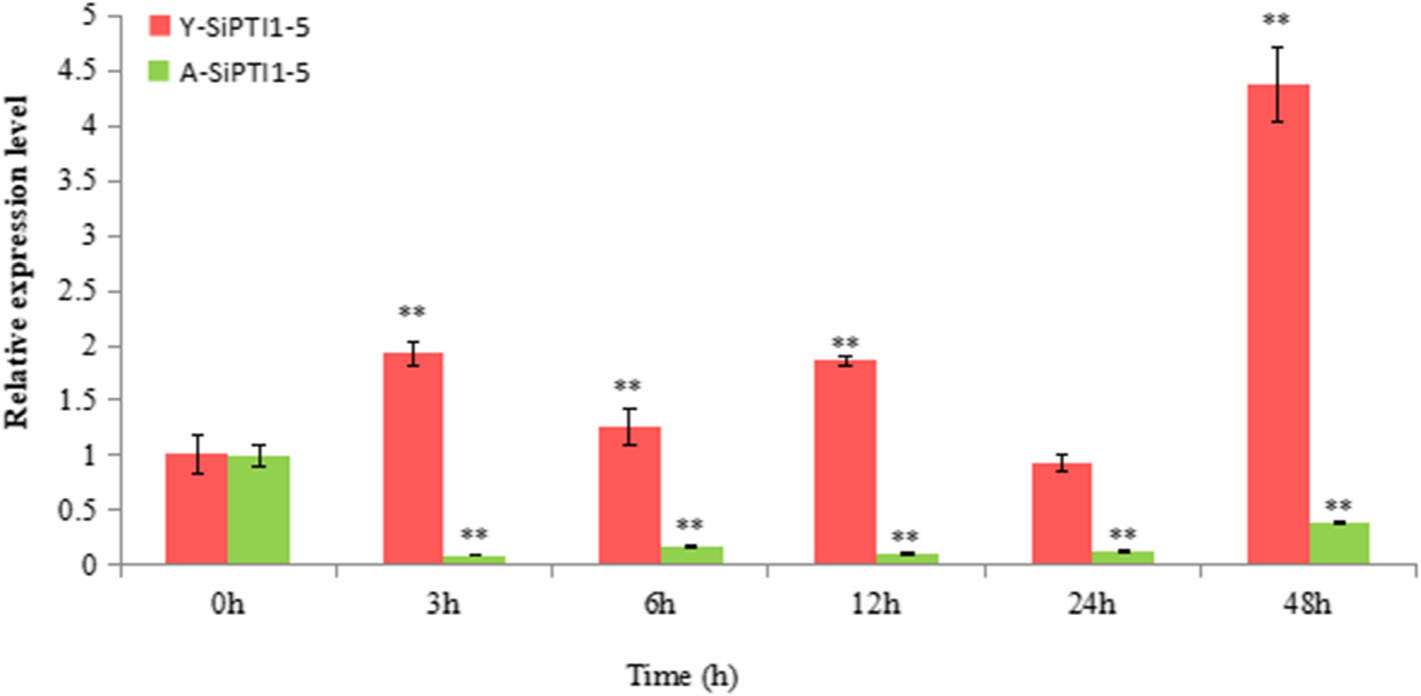
Expression pattern analysis of *SiPTI1–5* genes in different varieties of foxtail millet to salt stress treatments. Two-week-old seedlings of foxtail millet (‘Yugu1’, salt-tolerant variety, and ‘AN04’, a salt-sensitive variety are shown in red and green, respectively) leaves were treated with 150 mM NaCl. Transcription levels were analyzed via qRT-PCR and the expression of Y-*SiPTI1–5* (Represents the expression characteristics of *SiPTI1–5* in ‘Yugu1’)/A-*SiPTI1–5* (Represents the expression characteristics of *SiPTI1–5* in ‘AN04’), respectively, The values are the average of three biological repeats ± SD (standard deviation). Asterisks above bars denote a statistically significant difference by Duncan’s multi-range tests (*0.01 < P < 0.05, **P < 0.01)

In order to further evaluate the role of the *SiPTI1–5* in salt stress, the expression of *SiPTI1–5* gene was compared in ‘Yugu1’, salt-tolerant variety, and ‘AN04’, a salt-sensitive variety under salt (NaCl) treatment. The results showed that the expression of *SiPTI1–5* gene was up-regulated in ‘Yugu 1’, but down-regulated in ‘AN04’ (Fig. 10 and Additional file 9).

### Overexpression of *SiPTI1–5* in yeast conferred tolerance to salinity

**Fig. 11:**
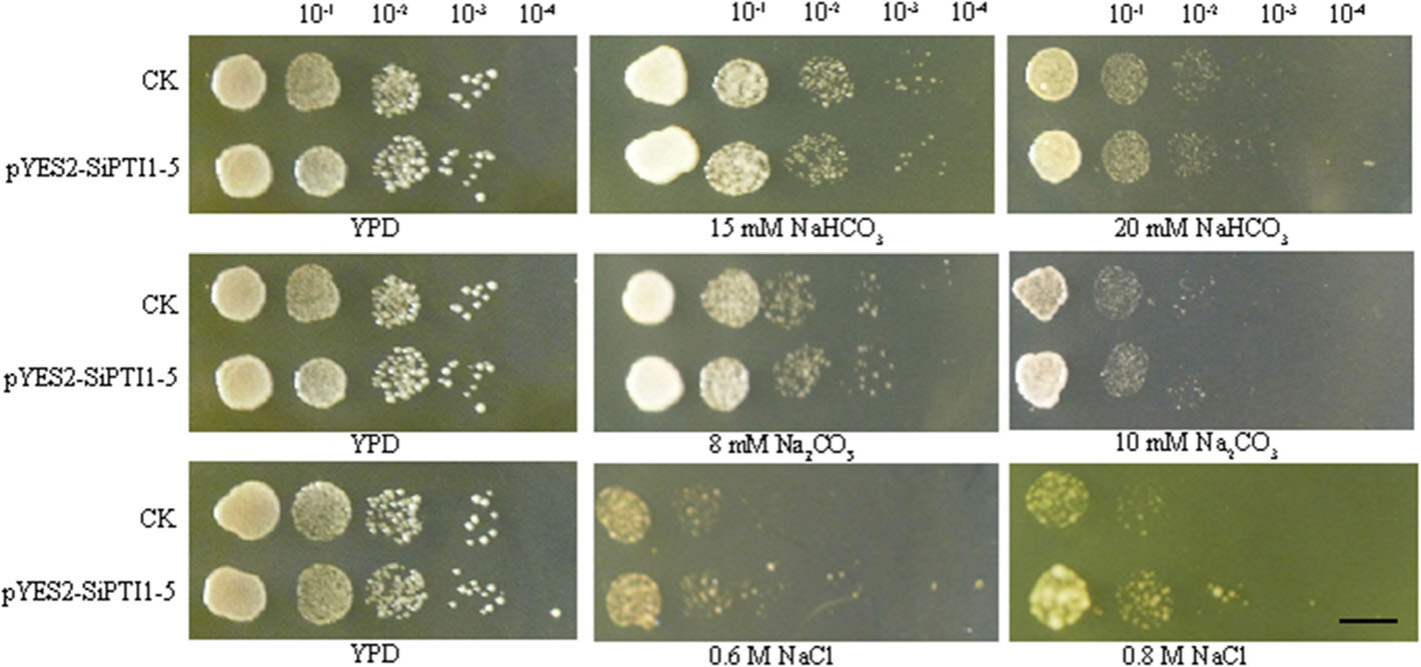
Assay for salt stress tolerance of *SiPTI1–5* transformed yeast. The pYES2-SiPTI1–5 fusion vectors were transformed into Invsc I yeast cells. The transformants were cultivated on YPD plates with NaHCO_3_, Na_2_CO_3_ and NaCl for two or three days. The 10^− 1^, 10^− 2^, 10^− 3^ and 10^− 4^ represent the dilution fold. Bar = 1 cm, transformant with empty vector pYES2 was used as a control (CK)

In the YPD medium without salt stress, there was almost no difference between control yeast strain (transformed with pYES2) and *SiPTI1–5*-expressing yeast strain (transformed with pYES2-SiPTI1–5) (Fig. 11). When exposed to Na_2_CO_3_ (8 mM, 10 mM) and NaHCO_3_ (15 mM, 20 mM) treatment, control strain and *SiPTI1–5*-expressing yeast strain had no difference in plaque growth at different concentrations, indicating that the *SiPTI1–5* does not confer tolerance to Na_2_CO_3_ and NaHCO_3_ stress in yeast, which is in agreement with the expression patterns of *SiPT1–5* in response to Na_2_CO_3_ and NaHCO_3_ (Fig. 11). There had shown no growth differences of control and *SiPTI1–5*-expressing yeast under 12 mM Na_2_CO_3_ and 25 mM NaHCO_3_ (data were not shown). Under NaCl stress, when NaCl concentration increased to 0.6 M, the SiPTI1–5-expressing yeast strain, grew better than the control strain (Fig. 11). In summary, the *SiPTI1–5* genes may be involved in response to salt stress induced by NaCl.

### Overexpression of *SiPTI1–5* in *E. coli* conferred tolerance to salinity stress

**Fig. 12:**
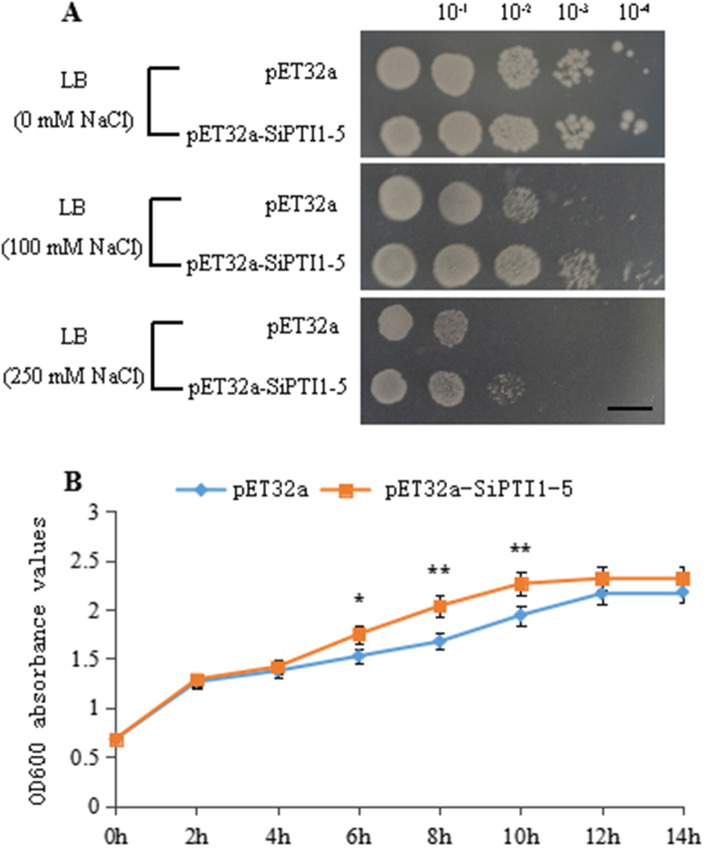
Assay for salt stress tolerance of *SiPTI1–5* transformed. The pET32a-SiPTI1–5 fusion vectors were transformed into *E. coli* (BL21) cells. The transformants were cultivated on LB plates with 0, 100 and 250 mM NaCl for 24 h. The 10^− 1^, 10^− 2^, 10^− 3^ and 10^− 4^ represent the dilution fold. Bar = 1 cm (**A**). Growth curves of pET32a-SiPTI1–5 plasmids containing BL21 strains in LB liquid medium with 250 mmol/L of NaCl. Transformant with empty vector pET32a was used as a control (**B**)

In order to test the relationship between SiPTI1–5 protein kinase and salt stress, the in vitro salt tolerance test was performed on control and *SiPTI1–5*-expressing strains (Fig. 12A). There are no significant differences in colony number between transformed *E. coli* harboring *SiPTI1–5* and the control under normal conditions, indicating that overexpression of *SiPTI1–5* did not affect the growth of *E. coli* recombinants in non-stress conditions. However, when grown on Luria-Bertani (LB) plates supplemented with 100 mM NaCl or higher, the number of transformed cells grew better than that of the control. Similar results were obtained in liquid LB with 250 mM NaCl, the growth rate of the *SiPTI1–5-*overexpressing strain was higher than that of the control strain, and it priorly arrived the logarithmic growth phase, indicating that the strain containing the pET32a-SiPTI1–5 recombinant plasmid had a certain salt-resistant ability compared with control (Fig. 12B).

These results demonstrated that overexpression of *SiPTI1–5* in *E. coli* was significantly enhanced tolerance to salt stress.

## Discussion

### Phylogenetic analysis revealed that *SiPTI1* genes were conserved in gramineous plant species

In this study, a total of 12 members of *PTI1* genes family were identified from foxtail millet. All the family members have the similar molecular wight and structure characteristics except *SiPTI1–5*. Most of PTI1s from various plant species contain about 300–400 amino acids (aa), while SiPTI1–5 contains 727 amino acids, and its molecular weight is about 81 kDa. Previous reports showed that most of the PTI1s were composed of 300–400 aa with a molecular weight of about 40 kDa, such as GmPTI1 (366 aa) of soybean [12], SlPTI1 (370 aa) of tomato [3], OsPTI1 (368 aa) of rice [14], and CsPTI1-L (362 aa) of cucumber [13]. Whether the larger SiPTI1–5 has specific function needs to be further investigated.

The phylogenetic analysis indicated that each SiPTI1 protein sequence was similar to their homologues from gramineous rice and maize. This implied that the orthologues proteins would share similar functions from a common ancestor [34]. It revealed the species bias in the distribution of the majority of foxtail millet *SiPTI1* genes in gramineous species, when compared to their homologues in dicot species. These were consistent with the present understanding of plant evolutionary history [35]. As a rational systematic approach, such phylogeny-based function prediction has been applied for prediction of stress-responsive proteins in other plant species such as rice [36] and maize [37].

New insights into the biological function of foxtail millet *PTI1* genes could be inferred by combining gene expression, phylogenetic and synteny analysis, as well as comparison with the function of known *PTI1* genes in model plant species. For example, *SiPTI1–5* exhibited the highest homology with its orthologs in rice OsNP_908680 (*OsPTI1b*) that mediates the hypersensitive response (HR), indicating that *SiPTI1–5* may share similar functions in foxtail millet. *SiPTI1–3* showed high degree of similarity with *ZmPTI1b* and *ZmPTI1a*, which implied that it probably be involved in flower development and defense stress [31, 38]. In addition, the multiple sequence alignment of PTI1 protein sequences implied that PTI1 were conserved among tomato, rice, maize, and foxtail millet. Especially, the kinase catalytic domain is highly conserved (Supplementary Fig. 2 and Supplementary Fig. 3).

We experimentally confirmed the predicted plasma membrane subcellular localization of SiPTI1–5 (Fig. 5). Interestingly, SiPTI1s lack predicted transmembrane structure or signal peptide. So, we speculated that its plasma membrane localization is due to interaction with the plasma membrane proteins [39]. Previous studies reported that rice OsPTI1a localizes to the plasma membrane through N-terminal palmitoylation and plays a role in immune responses via forming a complex at the plasma membrane [39]. As the phylogenetic tree branch shows that the *SiPTI1* gene family members of the foxtail millet had a closely relationship with the rice and maize, it is speculated that the mechanisms of action of foxtail millet *SiPTI1s* may be similar to rice and maize.

### The expression patterns of *SiPTI1s* under abiotic stresses

While essential for the growth and development of plants, excessive concentration of inorganic salts in the soil causes significant damage to the plants [40, 41], ranging from ion poisoning [42, 43], osmotic stress [44, 45], to oxidative stress [46, 47]. Salt stress is a prominent source of abiotic stress [48, 49] globally as over 20% of arable land and more than 40% of irrigated land [50, 51] worldwide considered to have some degree of excess salinity [52, 53]. Therefore, it is particularly important to study the salt tolerance mechanisms of plants, especially for agronomic crops.

In this study, qRT-PCR analysis revealed the expression characteristics of *SiPTI1* genes under diverse salinity treatments (150 mM NaCl, 100 mM NaHCO_3_, and 75 mM Na_2_CO_3_) (Fig. 9). Among twelve *SiPTI1* genes, the expression of *SiPTI1–5* was obviously induced under various treatments for 12 h. It is well known that salt stress is usually accompanied by excessive accumulation of ROS, including H_2_O_2_, that causes oxidative damage to proteins, DNA and lipids [54]. ROS are also involved in the regulation of cell proliferation [55], cell defense [56], and signal transduction [54, 57]. Therefore, oxidative stress is important for the study of the mechanisms of salt tolerance. Interestingly, the expression of *SiPTI1–5* was induced by NaCl and H_2_O_2_, indicating that *SiPTI1–5* participates in salt stress response through regulation ROS dynamic balance. It is well known that, the serine/threonine protein kinase OXI1, mediated oxidative stress signaling. Previous research report that *AtPTI1–2* could been activated by OXI1 in response to PA, H_2_O_2_, and flagellin [13]. In addition, *AtPTI1–4* signals via OXI1 and MPK6 signaling cascades functioned in oxidative stress [8]. Moreover, the *PTI1* genes also were induced by other stresses. For example, *GmPTI1* expression was induced by salicylic acid and wounding [12], *ZmPTI1–1* was dramatically induced by abscisic acid (ABA) and mannitol [58], and the *CsPTI1-L* of cucumber expression was induced when cucumber plants were challenged with the fungal pathogen *Sphaerotheca fuliginea* or with salt treatment [13]. It is well known that promoters could regulate temporal and spatial expression of gene, and *cis*-elements in promoters are crucial for gene function regulation by interacting with trans-acting factors. In this study, the promoters of *SiPTI1* family members were analyzed, and a large number of *cis*-elements related to stress response (e.g., MYB, MYC, ABRE, and DRE) were found. Meanwhile, the qRT-PCR results of the *SiPTI1* family members indicated that they could be induced by various salinity and oxidative stresses, which were well correlated with previous reports. Furthermore, the expressions of *SiPTI1–5* in ‘Yugu1’ and ‘AN04’ under salinity were analyzed (Fig. 10), which implied that the *SiPTI1–5* were positively correlated with salt stress response.

### *SiPTI1–5* gene is involved in salt tolerance

The *PTI1* gene was identified in tomatoes firstly, which was involved in a Pto-mediated signaling pathway, acting as a member downstream of *Pto* in a phosphorylation cascade during plant-pathogen interaction [3]. Besides, different members of PTI1 family have been reported to function in stress response in Arabidopsis [1, 8], soybean [12] and cucumber [13]. The *PTI1* gene in monocotyledonous maize [31], wheat [39, 59], and rice [39, 59] were involved in flower development and stress response, respectively. It has been reported that over-expression of *PTI1-like* gene *ZmPTI1* in Arabidopsis enhanced the salt resistance [38], and over-expression of *ZmPTI1–1* significantly enhanced the drought tolerance of Arabidopsis [58]. In addition, over-expression *CsPTI1-L* of cucumber positive regulated the responses of pathogen-defense and salt-stress [13]. In the current study, our results revealed that over-expression of *SiPTI1–5* genes in yeast and *E. coli*. Strains increased their salinity tolerances. Taken together with its stress induction, we speculated that *SiPTI1–5* genes would play important roles in foxtail millet in response to salinity and oxidative responses. However, the detailed salinity-responsive mechanism in the phosphorylation cascade needs to be further confirmed.

## Conclusion

A total of 12 putative *SiPTI1* genes were identified in foxtail millet using genome-wide analysis. The chromosomal distribution, intron-exon structures, motifs, duplication and divergence rates, *cis*-acting elements and subcellular localizations of the resulting proteins were analyzed. Synteny analysis and phylogenetic comparison of *PTI1* genes from several different plant species provided valuable clues about the evolutionary characteristics of foxtail millet *SiPTI1* genes. *SiPTI1* genes play important roles in foxtail millet growth and development, and the expression patterns showed that they are induced by various developmental and environmental cues. The phylogenetic and gene expression analysis shed some lights on the functional analysis of *SiPTI1* genes, suggesting a role for *SiPTI1–5* may be involved in salt tolerance. Heterologous expression of *SiPTI1–5* in yeast and *E. coli* enhanced tolerance to salt stress in this study. These results provide a valuable resource for better understanding of the biological roles of individual *SiPTI1* genes in foxtail millet.

## Methods

### Plant materials and growth conditions

The seeds of “Yugu1” and “AN04” were kindly provided by Professor Diao Xianmin, Chinese Academy of Agricultural Sciences, Beijing.

For organ expression analysis of *SiPTI1* genes, the seeds of “Yugu1” were soaked in water and germinated at 28 °C for two days, and then the seeds were sowed in the field and the seedlings were cultured. At florescence, the samples were collected from roots, sixth internode, the seventh leaf and its sheath, as well as flowers, respectively.

For stress-responsive analysis of *SiPTI1* genes, three-week-old seedlings cultured in Hoagland solution were exposed to various salinity treatments (150 mM NaCl, 75 mM Na_2_CO_3_, and 100 mM NaHCO_3_), as well as 10 mM H_2_O_2_ for 0 h, 2 h, 4 h, 6 h, 8 h, 10 h, 12 h, 24 h, 48 h, and 72 h. Besides, for the comparation of the *SiPTI1–5* gene expression in “Yugu1” (salt-tolerant variety) and “AN04” (salt-sensitive variety), two-week old seedlings from two varieties were cultured and treated with 150 mM NaCl for 0 h, 3 h, 6 h, 12 h, 24 h and 48 h. After treatments, the five young leaves were collected for qRT-PCR analysis.

In these experiments, collections from five plants were pooled in each sample, and the samples were frozen immediately in liquid nitrogen and then stored at − 80 °C for further analysis. For each sample, three biological replications were performed for qRT-PCR analysis.

### Total RNA isolation, cDNA synthesis and quantitative real-time PCR

The total RNA of foxtail millet was extracted by TransZol Up (TRANS), and the specific experimental steps were described in the instructions. RNA integrity has been confirmed by electrophoresis with 1% agarose gels. The expression characteristics of *SiPTI1s* in foxtail millet under different stress treatments were detected by qRT-PCR. For each plant sample, 1 μg of total RNA was reverse transcribed to cDNA in a 20 μl reaction system using a PrimeScriptTM 1st Strand cDNA Synthesis Kit (TaKaRa). The primers used for qRT-PCR analysis were designed from a non-conserved region by Primer-BLAST (http://www.ncbi.nlm.nih.gov/tools/primer-blast/) [34]. *SiActin* gene (AF288226.1) was used as reference gene for qRT-PCR analysis [34]. The primers used in these experiments are listed in the Additional file 8. Fold change was calculated using the 2^−ΔΔCt^ method [44]. Each experiment was repeated for three times. The data were shown as means ± standard deviation (SD). Statistical analysis was performed on SPSS 17.0. The statistical significance was determined using an analysis of variance (ANOVA), and significant differences (*P* < 0.05) between the values were determined using Duncan’s multiple range test [44].

### Bioinformatic analysis of the *SiPTI1* family in foxtail millet

A Hidden Markov Model (HMM) was established by indexing the *PTI1* family sequence of Rice, Arabidopsis, and Maize, and HMM profile was prepared using HMMER suite [60]. The HMM profile was then searched against the foxtail millet proteome data under default *E* value cut-off of 0.01 [61]. The sequences of *SiPTI1s* (coding sequences (CDS), Protein and Gene) were all downloaded from Phytozome (JGI) (https://phytozome.jgi.doe.gov/pz/portal.html), and demonstrate in Additional file 1, whereas, Arabidopsis and maize *PTI1* sequences (CDS, Protein and Gene) were deposited from Ensembl (http://plants.ensembl.org/index.html). Each putative *PTI1* gene sequence was checked against three databases: SMART (https://www.omicsclass.com/article/681), NCBI CDD (https://www.omicsclass.com/article/310), and Pfam (http://pfam.xfam.org/databas) to confirm the presence of the *PTI1* domain. The predicted genes were further validated by PCR amplification and sequencing, 12 *PTI1* genes models were finally identified in the foxtail millet genome after comprehensive curation, for nomenclature, the prefix ‘*Si*’ for *S. italica* was used, followed by ‘*PTI1*’, which were designated from *SiPTI1–1* through *SiPTI1–12* on the basis of their chromosomal location. Length of sequences, molecular weights, isoelectric points of identified PTI1 proteins were obtained using tools from ExPasy website (http://web.expasy.org/protparam/). In addition subcellular locations were predicted using five publicly available tools: http://abi.inf.uni-tuebingen.de/Services/YLoc/webloc.cgi, https://rostlab.org/services/loctree3/, http://www.csbio.sjtu.edu.cn/bioinf/plant-multi/, http://genome.unmc.edu/ngLOC/index.html, and http://www.cbs.dtu.dk/services/TargetP/ according to Suo et al. [62].

### Phylogenetic analysis of *PTI1* genes

To further investigate the evolutionary relationships of the PTI1 proteins in various plants species, the phylogenetic trees of the PTI1 was constructed. Multiple sequence alignment of PTI1 protein sequences were conducted with the ClustalX 1.81 program using the default multiple alignment parameters. The unrooted phylogenetic tree were constructed using MEGA7.0 software with a maximum likelihood method using sequences from *S. italica* (*Si*), *S. lycopersicum* (*Sl*), *N. tabacum*, (*Nt*), *A. thaliana* (*At*), *O. sativa* (*Os*), and *Z. mays* (*Zm*) [31], the PTI1 protein sequences used to construct phylogenetic tree but does not include SiPTI1s were acquired from NCBI (https://www.ncbi.nlm.nih.gov/) and the corresponding protein sequences of list in Additional file 2. The bootstrap consensus tree inferred from 1000 replicates [63, 64].

### Homologous alignment of PTI1 protein sequences

The sequences alignment analysis of PTI1s from foxtail millet, tomato, rice and maize. Was conducted using DNAMAN_6.0.

### Chromosomal location, gene structure analysis, promoter analysis and estimation of genomic distribution and gene duplication

All *SiPTI1* genes were mapped to the nine foxtail millet chromosomes according to their ascending order of physical position (bp), from the short arm telomere to the long arm telomere, and were visualized using MapChart [65]. The exon-intron structures of the *SiPTI1* genes were determined by comparing the CDS with their corresponding genomic sequences using the Gene Structure Display Server (GSDS) (http://gsds.cbi.pku.edu.cn/) [66]. The MEME online program (http://meme.nbcr.net/meme/ intro.html) for protein sequence analysis was used to identify conserved motifs in the identified foxtail millet PTI1 proteins [67]. The optimized parameters were employed are the following: the number of repetitions: any, the maximum number of motifs: 15, and the optimum width of each motif: between 6 and 100 residues [34, 68]. The cis-regulatory elements were identified using Plantcare (http://bioinformatics.psb.ugent.be/webtools/plantcare/html/) database. All Si*PTI1* genes were mapped to foxtail millet chromosomes based on physical location information from the database of foxtail millet genome using Circos [69]. Multiple Collinearity Scan toolkit (MCScanX) adopted to analyze the gene duplication events, with the default parameters [33, 70]. To exhibit the synteny relationship of the orthologous *PTI1* genes obtained from foxtail millet and other selected species, the syntenic analysis maps were constructed using the Dual Systeny Plotter software (https://github.com/CJ-Chen/TBtools) [71]. Non-synonymous (ka) and synonymous (ks) substitution of each duplicated *PTI1* genes were calculated using KaKs_Calculator 2.0 [72, 73]. Substitution rate of the *PTI1* genes Ks and Ka were estimated according to previously-described criteria [34, 74] Ks and Ka substitution rates were calculated using the CODEML program and confirmed with the GEvo tool (https://genomevolution.org/CoGe/SynMap.pl). The time (million years ago, MYA) of duplication and divergence time (T) was calculated using a synonymous mutation rate of λ substitutions per synonymous site per year as T = Ks/2λ (λ = 6.5 × 10–9) [33].

### Subcellular localization of SiPTI1–5

The recombinant plasmid pBI121-SiPTI1–5-GFP was generated by amplifying the coding sequence of SiPTI1–5 without the termination codon, and then inserting the sequence into the *Xba*I/*Sal*I restriction site of pBI121-GFP. Onion epidermal cells were bombarded with the constructs pBI121-GFP and pBI121-SiPTI1–5-GFP, and used a particle gun-mediated system PDS-1000/He (Bio-Rad, Hercules, CA, USA). GFP signals were observed with a confocal laser scanning microscopy (LSM 510, Carl Zeiss MicroImaging GmbH, Jena, Germany) [75].

### Assay for salinity tolerance of *E. coli* transformants

The recombinant plasmid pET32a-SiPTI1–5 was generated by amplifying the coding sequence of *SiPTI1–5* without the termination codon, and then inserting the sequence into the *Sac*I/*Xho*I restriction site of pET32a. Then pET32a empty vector (as control) and pET32a-SiPTI1–5 recombinant plasmid were transformed into *E. coli* host strain BL21 (DE3), respectively. The expression of *SiPTI1–5* in the recombinant cells was confirmed by SDS-PAGE analysis (Supplementary Fig. 1). Transformed *E. coli* BL21 (DE3) cells carrying pET32a-SiPTI1–5 or pET-32a were grown overnight in LB liquid medium (contained 100 μg/ml ampicillin), respectively, which culture condition was 37 °C, 180 rpm. For salinity resistance analysis, the bacterial cultures were diluted 50-fold using liquid LB, and incubated for 2–3 h at 37 °C until OD600 = 0.5–0.6 [75]. Isopropylthio-β-D-galactoside (IPTG) was added to the cultures and make it final concentration was 0.5 mM for induction of expression of the inserted gene.

Spot assay was applied for salinity resistance analysis of *SiPTI 1–5* transformed *E. coli* [75]. After 4 h (25 °C) IPTG induction, the concentration of *E. coli* was adjusted to OD600 0.6 using LB liquid medium (contained 100 μg/ml ampicillin) [75]. In order to measure the response to salinity, the samples were diluted by 10^− 1^, 10^− 2^, 10^− 3^, and 10^− 4^ folds with LB medium contained ampicillin. Three microliters of each diluted sample were plated on LB agar plates, LB agar plates supplemented with 0, 100, 250 mM NaCl, respectively. After incubation for 12 h on LB agar plates at 37 °C [75]. The bacterial colony growth under salt stress was recorded with Canon digital camera.

For salt resistance detection of *SiPTI 1–5* transformed *E. coli* in liquid culture media, the bacteria were cultured for 14 h at 25 °C in liquid LB after IPTG induction. The absorbance value at OD600 was measured every 2 h and the data were recorded until OD600 reached to approximately two. The experiments were repeated for three times.

### Assay for salt-stress tolerance of yeast transformants

The sequence of *SiPTI1–5* was amplified and cloned into the *Kpn*I/*Xho*I sites of pYES2 to construct the expression vector pYES2-SiPTI1–5, which was then transformed into *y*east host strain INVSc 1. The pYES2 empty vector was used as the control. Fresh cultures of control and pYES2-SiPTI1–5 strains were prepared and adjusted to OD600 of 0.6 in YPD medium. This culture was successively diluted to 10^− 1^, 10^− 2^, 10^− 3^, 10^− 4^ times, spotted on YPD (No salinity) or YPD medium supplemented with Na_2_CO_3_ (8 mM, 10 mM, and 12 mM), NaHCO_3_ (15 mM, 20 mM, and 25 mM), or NaCl (0.6 M, 0.8 M, and 1 M) and incubate at 28 °C for 2 days to observe and photograph the phenotype with Canon digital camera. All experiments were repeated independently for three times.

## Supplementary Information

**Additional file 1. **The sequences of *PTI1* genes in foxtail millet.

**Additional file 2.** The PTI1 proteins used to construct phylogenetic but does not include SiPTI1s.

**Additional file 3.** Detailed characteristics of the motifs in the SiPTI1 proteins.

**Additional file 4. **The specific location of each *SiPTI1* gene on the chromosomes.

**Additional file 5. **Characteristics of the promoter region of *SiPTI1* genes.

**Additional file 6. **Segmentally and tandemly duplicated *SiPTI1* gene pairs.

**Additional file 7.** One-to-one orthologous relationships between foxtail millet and other two plant species.

**Additional file 8.** Sequences of the primers used in this study.

**Additional file 9. **The relative expression value of *SiPTI1s*.

**Additional file 10: Supplementary Fig. 1.** SiPTI1–5 fusion protein identification by SDS-PAGE electrophoresis. M: marker, 1: pET32a (0 h), 2: pET32a-SiPTI1–5 (0 h), 3: pET32a-SiPTI1–5^T604A^ (0 h), 4: pET32a-SiPTI1–5^K452N^ (0 h), 5: pET32a (4 h), 6: pET32a-SiPTI1–5 (4 h), 7: pET32a-SiPTI1–5^T604A^ (4 h), 8: pET32a-SiPTI1–5^K452N^ (4 h).

**Additional file 11: Supplementary Fig. 2.** Sequence homology of SiPTI1s. The sequences alignment of PTI1s from foxtail millet and tomato.. The 11 canonical subdomains conserved in serine/threonine kinases are indicated with Roman numerals. Invariant residues common to the majority of protein kinases are marked with black dots. The highly conserved lysine residue in subdomain II which is required for activity in SlPTI1 and most protein kinases is boxed.

**Additional file 12: Supplementary Fig. 3.** Sequence homology of PTI1s. The sequences alignment of PTI1s from foxtail millet, rice and maize. The 11 canonical subdomains conserved in serine/threonine kinases are indicated with Roman numerals. Invariant residues common to the majority of protein kinases are marked with black dots. The highly conserved lysine residue in subdomain II which is required for activity in most protein kinases is boxed.

## Data Availability

All relevant data of this article are available within the manuscript and its additional files. The sequences of *SiPTI1s* (coding sequences (CDS), Protein and Gene) were all downloaded from Phytozome (JGI) (https://phytozome.jgi.doe.gov/pz/portal.html), and demonstrated in Additional file 1, whereas, Arabidopsis and maize *PTI1* sequences (CDS, Protein and Gene) were deposited from Ensembl (http://plants.ensembl.org/index.html). The PTI1 protein sequences used to construct phylogenetic tree but does not include *SiPTI1s* were acquired from NCBI (https://www.ncbi.nlm.nih.gov/) and the corresponding protein sequences of list in Additional file 2.

## Abbreviations

RLK: Receptor-like protein kinases
PTI1: Pto-interacting 1
CDS: Coding sequence
ORF: Open reading frame
qRT-PCR: Quantitative real-time PCR
UTR: Untranslated region
ABA: Abscisic Acid
MeJA: Methyl Jasmonate
PEG: Polyethylene Glycol
SA: Salicylic acid
IPTG: Isopropylthio-β-D-galactoside
LB: Luria-Bertani
OXI1: Oxidative signal-inducible 1
ROS: Reactive oxygen species
MAPK: Mitogen-activated protein kinase
