# Supplementary material for: Genome-wide identification of PTI1 family in Setaria italica and salinity-responsive functional analysis of SiPTI1–5
Source: BMC Plant Biol. 2021 Jul 3;21:319. doi: 10.1186/s12870-021-03077-4 (PMC8254068; doi:10.1186/s12870-021-03077-4)

**Article title:** Genome-wide Identification of *PTI1* Family in *Setaria italica* and Salinity-responsive Functional Analysis of *SiPTI1-5*

**Author list:** Yongguan Huangfu^1^, Jiaowen Pan^2^, Zhen Li^2^, Qingguo Wang^2^, Fatemeh Mastouri^6^, Ying Li^1^, Stephen Yang^7^, Min Liu^5^, Shaojun Dai^3*^ and Wei Liu^2,4*^

**Detailed characteristics of the motifs in the SiPTI1 proteins**

Motif 1


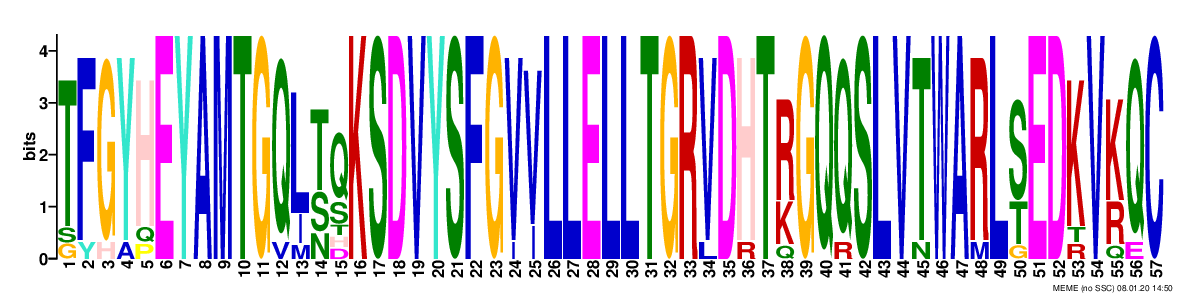


Motif 2


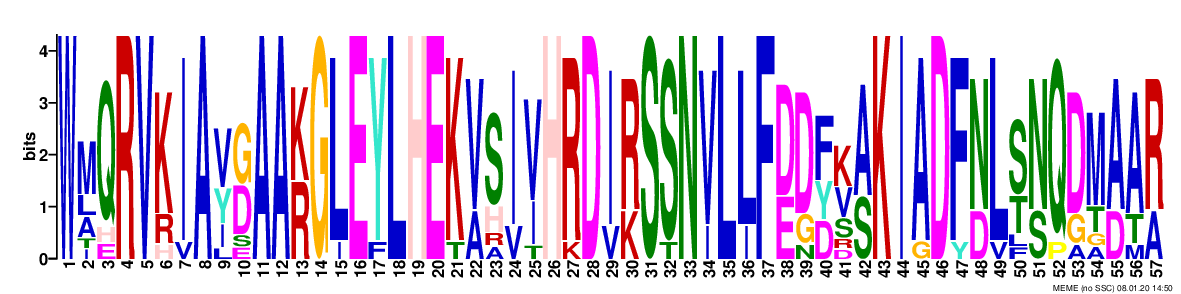


Motif 3


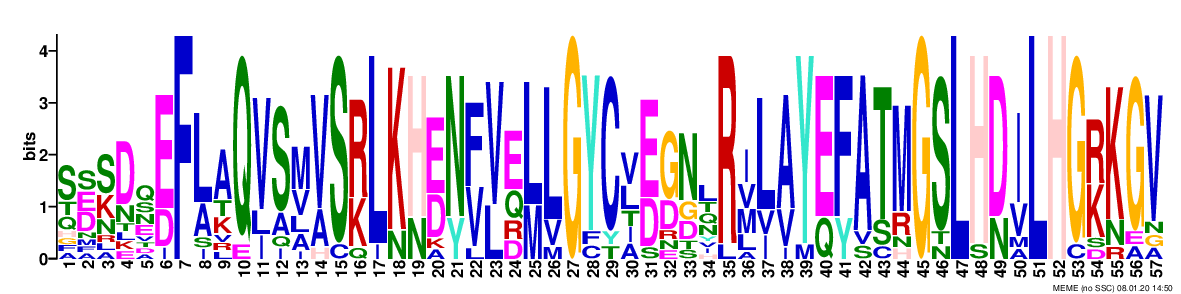


Motif 4


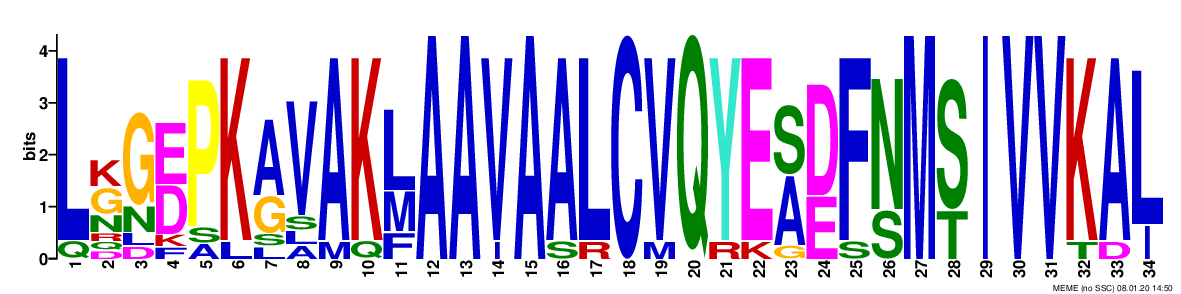


Motif 5


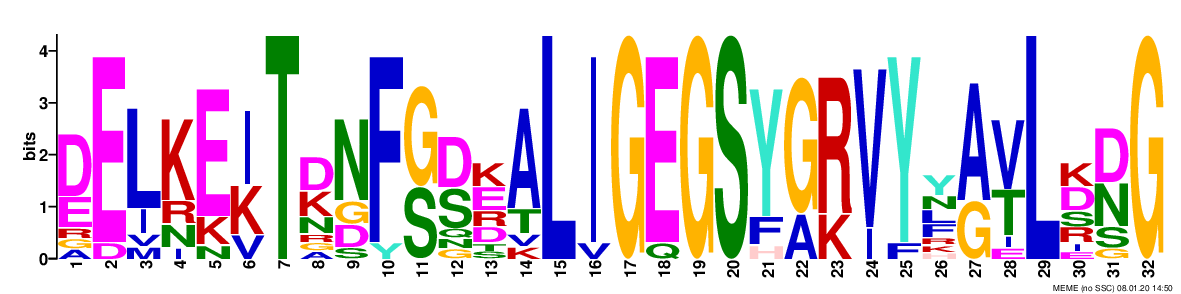


Motif 6


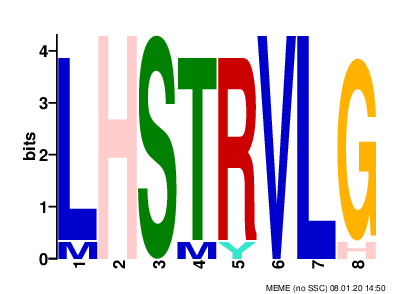


Motif 7


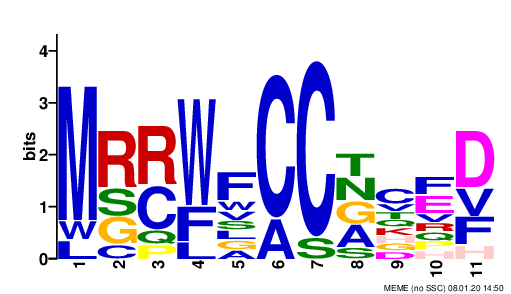


Motif 8


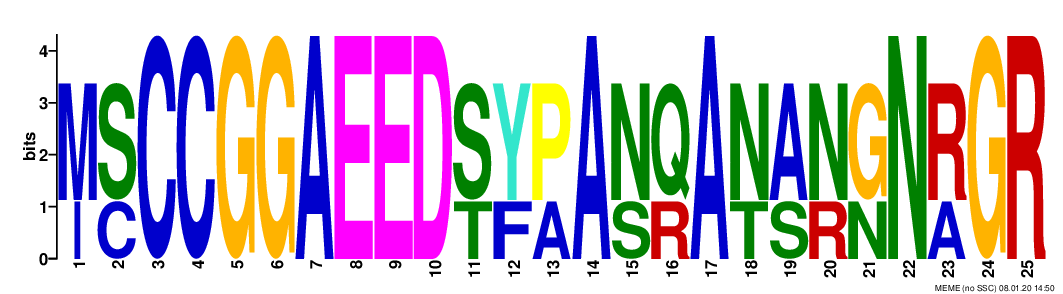


Motif 9


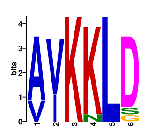


Motif 10


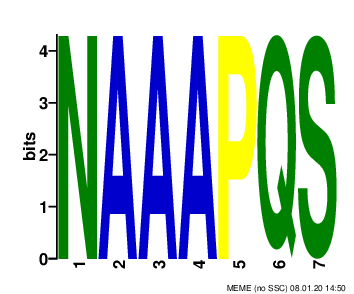

Supplement: Supplementary file 3 — Additional file 3. Detailed characteristics of the motifs in the SiPTI1 proteins. [file 12870_2021_3077_MOESM3_ESM.doc]
