# Supplementary figures and images for: Genome-wide identification of PTI1 family in Setaria italica and salinity-responsive functional analysis of SiPTI1–5
Source: BMC Plant Biol. 2021 Jul 3;21:319. doi: 10.1186/s12870-021-03077-4 (PMC8254068; doi:10.1186/s12870-021-03077-4)

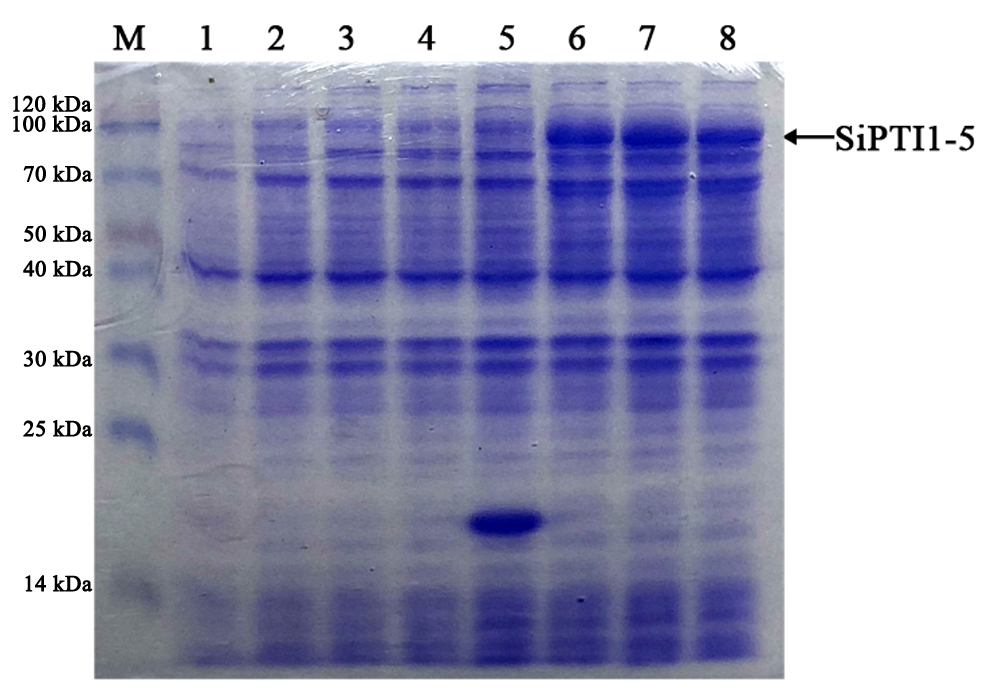

Supplement: Supplementary file 10 — Additional file 10: Supplementary Fig. 1. SiPTI1–5 fusion protein identification by SDS-PAGE electrophoresis. M: marker, 1: pET32a (0 h), 2: pET32a-SiPTI1–5 (0 h), 3: pET32a-SiPTI1–5T604A (0 h), 4: pET32a-SiPTI1–5K452N (0 h), 5: pET32a (4 h), 6: pET32a-SiPTI1–5 (4 h), 7: pET32a-SiPTI1–5T604A (4 h), 8: pET32a-SiPTI1–5K452N (4 h). [file 12870_2021_3077_MOESM10_ESM.jpg]

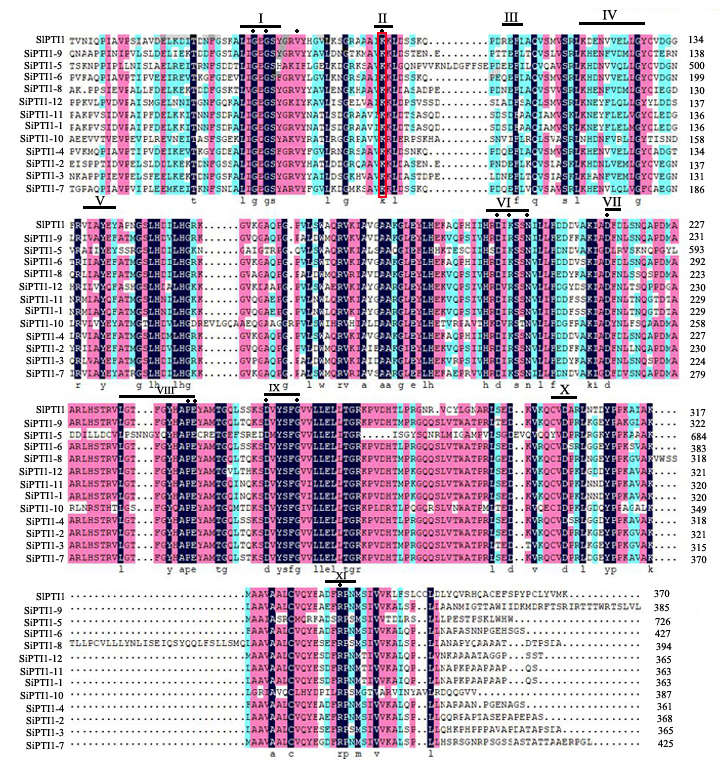

Supplement: Supplementary file 11 — Additional file 11: Supplementary Fig. 2. Sequence homology of SiPTI1s. The sequences alignment of PTI1s from foxtail millet and tomato.. The 11 canonical subdomains conserved in serine/threonine kinases are indicated with Roman numerals. Invariant residues common to the majority of protein kinases are marked with black dots. The highly conserved lysine residue in subdomain II which is required for activity in SlPTI1 and most protein kinases is boxed. [file 12870_2021_3077_MOESM11_ESM.jpg]

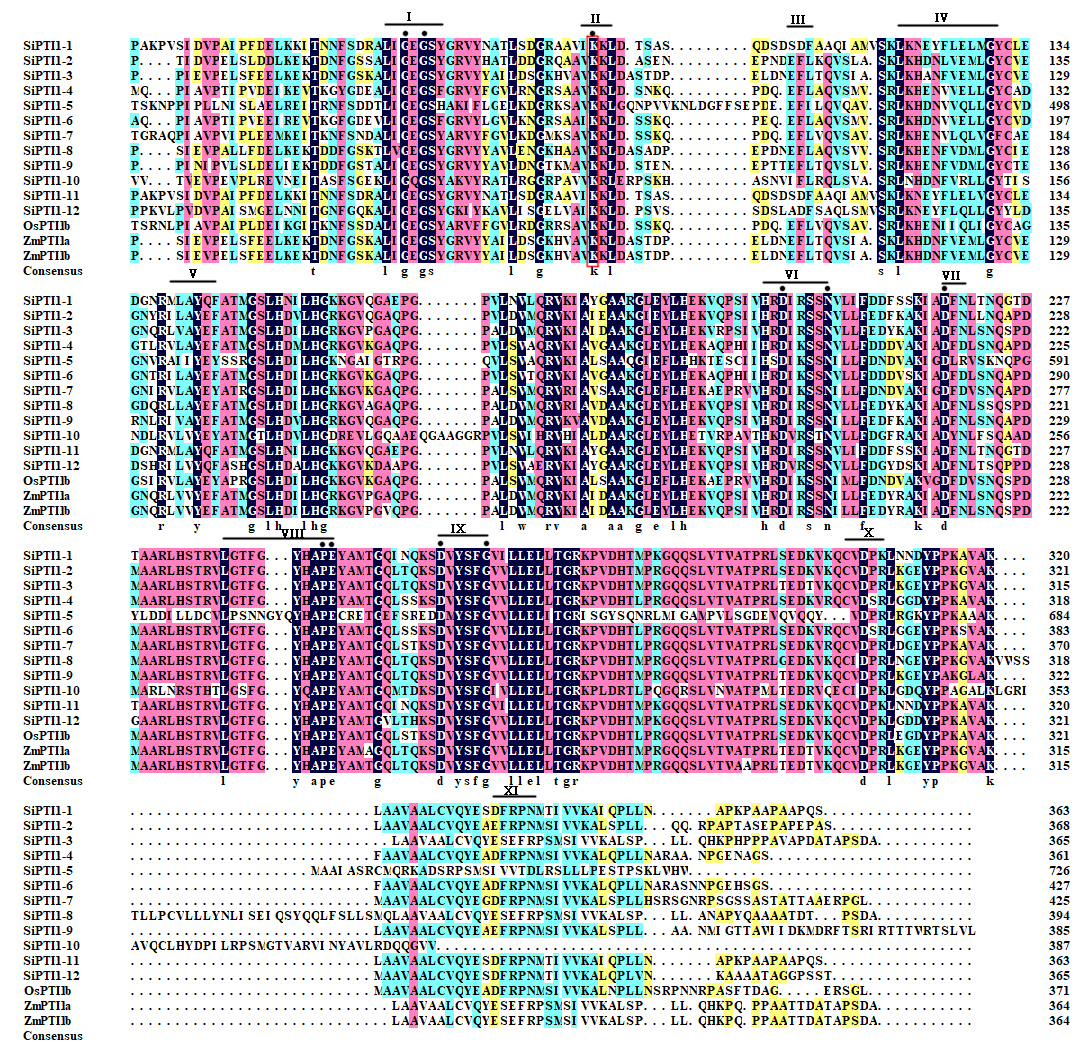

Supplement: Supplementary file 12 — Additional file 12: Supplementary Fig. 3. Sequence homology of PTI1s. The sequences alignment of PTI1s from foxtail millet, rice and maize. The 11 canonical subdomains conserved in serine/threonine kinases are indicated with Roman numerals. Invariant residues common to the majority of protein kinases are marked with black dots. The highly conserved lysine residue in subdomain II which is required for activity in most protein kinases is boxed. [file 12870_2021_3077_MOESM12_ESM.jpg]
